# Self-control, academic anxiety, and mobile phone addiction: the moderating role of being an only child

**DOI:** 10.3389/fpsyg.2025.1587279

**Published:** 2025-06-13

**Authors:** De Su, Jiahao Zhang, Yuanyuan Ma, Ze Geng

**Affiliations:** ^1^School of Education, Minzu University of China, Beijing, China; ^2^Institute for Research on the High-Quality Development Strategy of Education in Ethnic Minority Areas, Minzu University of China, Beijing, China

**Keywords:** mobile phone addiction, academic anxiety, self-control, only-child status, middle school students

## Abstract

**Background:**

The prevalence of mobile phone addiction among adolescents is a growing concern with significant implications for psychological well-being and academic performance. The mediating role of academic anxiety (AA) in the relationship between self-control (SC) and mobile phone addiction (MPA) among middle school students deserves thorough investigation, particularly considering the significant moderating effect of Being an only child on these relationships.

**Methods:**

A cross-sectional survey of 2,489 middle school students (1,257 girls and 1,232 boys) assessed SC, AA, and MPA. SC was measured using the Self-Control Scale, AA with the Academic Anxiety Scale (AAS), and MPA with the Mobile Phone Addiction Index (MPAI). Structural equation modeling analyzed the mediating and moderating effects.

**Results:**

Self-control significantly negatively predicted AA (*β* = −0.464, *p* < 0.001) and MPA (*β* = −0.563, *p* < 0.001). AA was identified as a significant mediator that positively predicted MPA (*β* = 0.173, *p* < 0.001) and mediated the relationship between SC and MPA (*β* = 0.081, 95%CI = [−0.10, −0.06]). The moderating effect of being an only child on the relationship between AA and MPA (*β* = −0.13, *p* < 0.001) was significant.

**Conclusion:**

The empirical evidence substantiates the mediating role of AA in the relationship between SC and MPA, while simultaneously demonstrating that only children exhibit heightened susceptibility to MPA with increasing AA levels. Such observations significantly advance our understanding of the influence of family dynamics on MPA manifestation among adolescents.

## 1 Introduction

With the advancement of communication technology, smartphones have become essential in our daily lives, providing unparalleled convenience for personal activities, professional tasks, and educational pursuits. As of June 2024, China added 7.42 million new internet users, of whom 99.7% accessed the internet via mobile phones, with 49% being adolescents, making them the primary force among new mobile internet users (The 54th Statistical Report on China’s Internet Development, 2024). However, inappropriate smartphone usage habits can lead to smartphone addiction behaviors (Xie et al., 2019). Mobile phone addiction (MPA), commonly referred to as “smartphone behavioral addiction” (Shambare et al., 2012), “mobile phone dependence” (Chóliz, 2012), or “problematic mobile phone use” (Lopez-Fernandez et al., 2014), refers to impaired social functioning caused by individuals’ excessive and uncontrollable smartphone use. Research indicates that adolescents are more susceptible to smartphone addiction (De-Sola et al., 2016). This addictive behavior interferes with adolescents’ physical and mental health, manifesting as poor academic performance, social isolation, and communication avoidance, leading to more serious social and interpersonal problems (Yang et al., 2019). Although previous research has demonstrated that individuals with stronger self-control (SC) are adept at managing their mobile phone use, thereby effectively reducing or eliminating addictive behaviors (Özdemir et al., 2014; Servidio, 2019), research on the mechanisms by which SC reduces smartphone addiction remains insufficient.

Middle school represents a critical stage in adolescents’ physical and psychological development (Wolfe et al., 2003). Adolescents are at a pivotal developmental juncture transitioning from psychological immaturity to maturity, making their emotional states susceptible to external influences that generate negative emotions (Olson et al., 2022). Mobile devices, serving as virtual platforms integrating entertainment, social networking, and other functions, have become outlets for adolescents’ negative emotional expression (Ji et al., 2025). Research demonstrates that psychological anxiety among adolescents has a significant positive impact on MPA (Servidio, 2019). However, enhancing SC capacity can effectively reduce negative emotions such as anxiety and depression, thereby preventing numerous problematic behaviors and mental health issues (Sinha, 2009). Existing research predominantly focuses on trait psychological anxiety, with limited attention to the impact of academic anxiety (AA) on MPA among adolescents. In reality, a considerable portion of adolescents’ psychological anxiety stems from AA caused by others’ academic expectations (Danli et al., 2024), which is particularly pronounced among Chinese students (Jin et al., 2020). When individuals experience AA, they may employ avoidant coping strategies (i.e., MPA) to alleviate real-world problems (Arslan, 2017) and reduce academic-related psychological distress (Chiu, 2014). However, when individuals possess strong SC, they can effectively suppress negative emotions, thereby reducing MPA (Hyoungkoo et al., 2012). Therefore, adolescents’ SC may inhibit their MPA by reducing their AA.

Furthermore, MPA behaviors among middle school students are frequently influenced by familial, educational, and social domains (Kim et al., 2018; Tesser, 1980; Rolan and Marceau, 2018). As the social unit most closely connected to secondary school students, the family’s influence is particularly significant. Existing investigations have explored the mechanisms by which family environment and parent–child relationships affect adolescents’ MPA (Kim et al., 2018), yet sibling relationships, as a fundamental aspect of family dynamics, are often overlooked (Rolan and Marceau, 2018). Research indicates that the number of children can significantly influence parental caregiving approaches (Nicole and Judith, 2010). In only-child families, parents place all their hopes on a single child, resulting in stricter supervision and higher educational expectations (Li et al., 2020), which trigger severe AA (Yang et al., 2025). Due to the absence of emotional support and psychological sharing from siblings, only children may often rely more heavily on smartphone use to alleviate AA (Lv et al., 2023). Several MPA studies have also demonstrated that adolescents from only-child families exhibit significantly higher levels of mobile phone dependence compared to adolescents from families with siblings (Xie et al., 2024). Consequently, adolescents from only-child families may be more susceptible to MPA as their AA levels increase.

**Figure 1:**
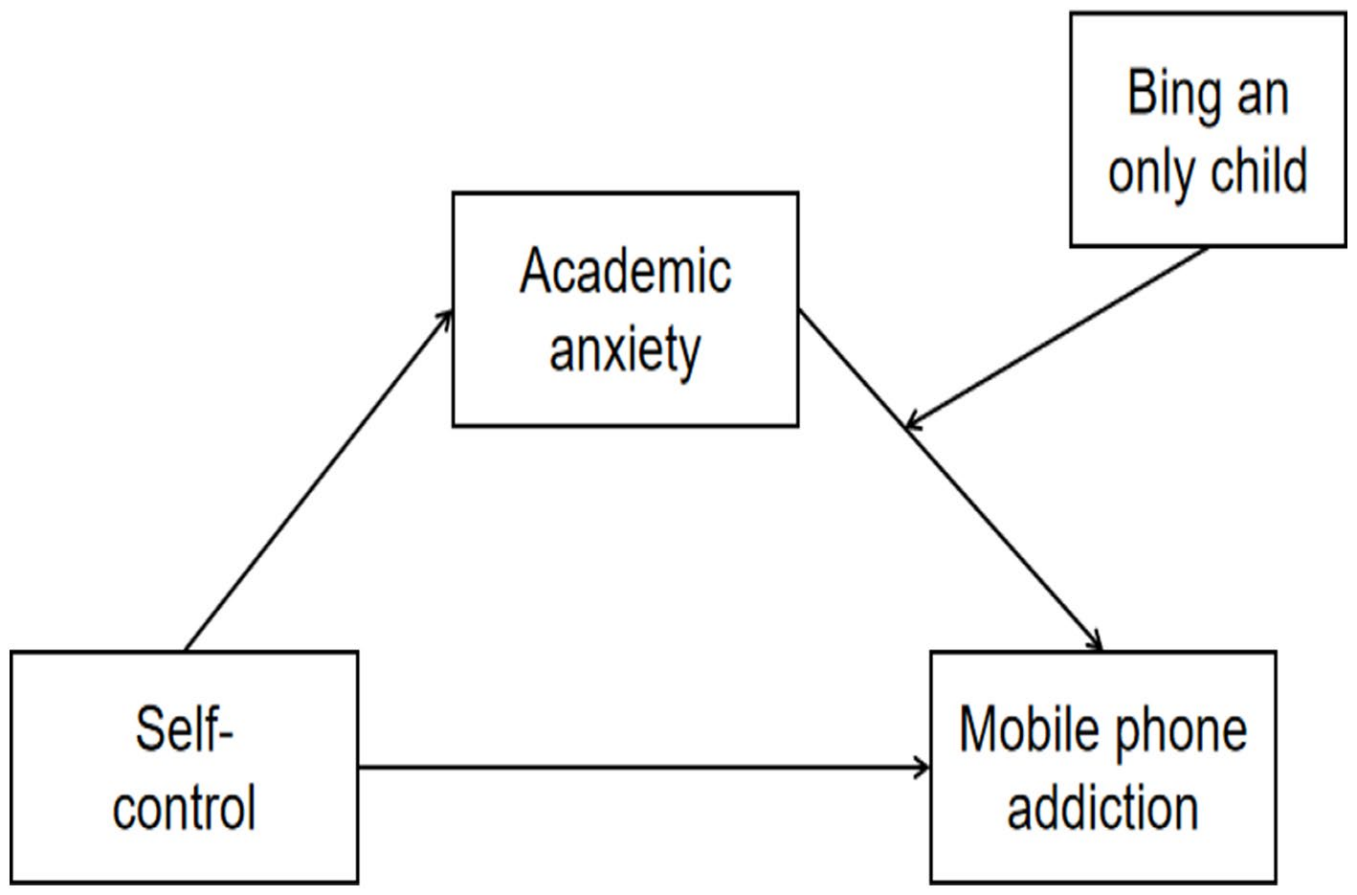
The conceptual model.

In summary, this study aims to investigate whether adolescents’ SC can suppress their AA and thereby weaken their MPA, and whether only child demonstrate higher MPA tendencies compared to non-only child when confronting AA. This research seeks to enrich mechanistic studies on how adolescents’ SC affects MPA and further expand research on family environment’s influence on adolescents’ problematic behaviors. Based on this foundation, the present study proposes the following research hypotheses and constructs a theoretical framework (see Figure 1):

*H1*: SC is inversely associated with MPA.

*H2*: SC is inversely associated with AA.

*H3*: AA mediates the relationship between SC and MPA.

*H4*: Being an only child has a direct effect on MPA.

*H5*: Being an only child will moderate the relationship between AA and MPA.

## 2 Method

### 2.1 Study design and setting

This study employed a cross-sectional design using self-report questionnaires. Data were collected online through convenience sampling from August to November 2024, targeting middle school students in China.

### 2.2 Participants

Participants were middle school students (grades 7–12) from Guangdong, Shandong, Zhejiang, and Shaanxi provinces in China. All participants were registered in the formal education system. Participation was voluntary and responses were anonymous.

### 2.3 Measures

#### 2.3.1 Self-control scale

The Self-Control Scale (SCS) was developed by Taney et al. (2004) and revised by Tan and Guo (2008), Cronbach’s coefficient of Tan’s study was 0.862, it is highly adaptable to Chinese culture. The scale comprises 19 items (For instance, “I am good at resisting temptation,” “I struggle to overcome bad habits.” “I often feel lazy,” and “I partake in activities that are detrimental to my well-being if they are enjoyable.”). Items 2, 3, 4, 6, 7, 8, 9, 10, 12, 13, 15, 16, 17, 18, and 19 are reverse-scored. A five-point scale was used, consisting of the following points: 1 for “strongly disagree,” 2 for “disagree,” 3 for “neutral,” 4 for “agree,” and 5 for “strongly agree.” Higher scores reflected greater levels of self-control, conversely, lower scores denoted a lack of self-control. Confirmatory factor analysis (CFA) demonstrated that the scale provided a good fit to the data (*χ^2^/df* = 6.54, RMSEA = 0.047, NFI = 0.925, TLI = 0.928). Cronbach’s coefficient of the SCS for middle school students in this study was 0.90.

#### 2.3.2 Academic anxiety scale

Academic anxiety was measured using the Academic Emotion Questionnaire (AEQ), developed by Perun et al. (2002) and further revised by Dong and Yu (2007), Cronbach’s coefficient of Dong’s study was 0.833, it shows great capacity to adapt to Chinese culture. The academic anxiety section includes seven items (e.g., “I feel nervous before exams,” “I worry that my grades will be worse than others,” “Sometimes I feel like I’m letting my family and teachers down because of my poor academic performance”). A five-point scoring system was used, where a lower score indicated a lower level of academic anxiety, and a higher score represented a higher level of academic anxiety. CFA demonstrated that the scale provided a good fit to the data (*χ^2^/df* = 4.89, RMSEA = 0.04, NFI = 0.981, TLI = 0.977). Cronbach’s coefficient of AA for middle school students in this study was 0.80.

#### 2.3.3 Mobile phone addiction index

The degree of smartphone addiction among study participants was assessed using the Mobile Phone Addiction Index (MPAI). Further, Leung (2008) developed the scale to assess the degree of cell phone dependence among adolescents and college students, Cronbach’s coefficient of Leung’s study was 0.90, it has a very good adaptability to Chinese culture. It comprises four dimensions: withdrawal, lack of control, inefficiency, and escapism, with 17 items (e.g., “You have tried to reduce the time you spend on your phone but have been unsuccessful; someone has mentioned that you spend too much time on it; you have attempted to hide your phone usage from others; and your phone bill exceeds your budget.”). The mobile phone questionnaire uses a five-point scoring system, with a higher score indicating a stronger tendency toward cell phone addiction. CFA demonstrated a good fit for the data (*χ^2^/df* = 7.68, RMSEA = 0.052, NFI = 0.952, TLI = 0.952). Cronbach’s coefficient of the MPA for middle students in this paper was 0.93.

### 2.4 Procedure and ethical considerations

Data were collected through convenience sampling using the Chinese Wenjuanxing platform^1^. Researchers distributed questionnaire links through WeChat Moments and class groups to invite eligible participants to voluntarily complete the survey. An informed consent section was placed on the front page of the questionnaire to ensure anonymous participation. To ensure data validity, responses were excluded based on the following criteria: (I) participants must have completed the questionnaire in at least 300 s, (II) questionnaires with excessive response consistency across scales were eliminated, (III) all participants must be registered in the formal education system, and (IV) one IP address was allowed to respond only once. Following these screening procedures, 2,489 valid cases were retained from the initial 3,578 collected questionnaires, representing an effective response rate of approximately 70%.

The research involving human subjects was approved by the Ethics Committee of Minzu University of China (approval number: MUC202410003). The study was conducted in strict compliance with the Declaration of Helsinki, local regulations, and institutional requirements. Additionally, this study’s questionnaires were completed anonymously, with a prominent notice on the first page stating: “If you fill out this questionnaire, it means that you are willing to allow the results of your responses to be used for scientific research. Otherwise, please exit the filling process. This survey is only for scientific research purposes, and participants’ privacy information will be strictly confidential.” Since the research subjects involved in this study are middle school students, and the survey questionnaires were distributed online, the questionnaires were initially sent to the mobile phones of the students’ guardians. Therefore, the collected questionnaire data was obtained with the consent of both the participants and their guardians.

### 2.5 Statistical analysis

The final data sample included in the study had no missing values, therefore no special treatment was required. Additionally, considering that this study explored two groups—only children and non-only children—we tested the measurement invariance of the model using AMOS 29.0 software during formal data analysis. The participants were divided into two groups: only child and non-only child. First, an unconstrained model M1 (Unconstrained) was constructed, then M2 (Measurement weights) was constructed on the basis of M1, and M3 (Measurement intercepts) was constructed on the basis of M2. The results showed that the fit indices of the three models all reached an acceptable level. The model comparison results indicated that the differences in fit indices between each pair of models (ΔCFI, ΔTLI, ΔNFI, ΔIFI, ΔRMSEA, ΔSRMR, all < 0.1), suggesting that the equivalence among the models holds. The model in this study has the same meaning and latent structure for both only child and non-only child.

Subsequently, formal analysis was conducted. First, SPSS Windows software version 26 was used to obtain descriptive statistics for all necessary variables and conduct Pearson correlation analyses. Second, PROCESS version 4.3 (Model 4) was utilized to examine the simple mediation model, while Model 14 was applied to assess the moderated mediation. The significance of the indirect and moderated mediation effects was assessed using a bootstrap sample of 5,000 with a 95% bias-corrected confidence range. The findings demonstrated the relevance of both indirect and moderated mediation effects by showing that the 95% bias-corrected confidence interval did not include zero (Preacher and Hayes, 2008). Demographic variables such as sex and grade were controlled in advance to eliminate the interference of irrelevant variables in the model analysis.

## 3 Results

### 3.1 Demographic characteristics

The final valid sample consisted of 2,489 students. Table 1 shows the demographic statistical description of the final valid samples. Of these, 1,257 (50.5%) were girls. In terms of grade distribution, there were 593 (23.8%) junior high school freshmen, 561 (22.5%) second-year students, 568 (22.8%) third-year students, 282 (11.3%) senior high school freshmen, 247 (9.9%) second-year students, and 238 (9.6%) third-year students. The sample included 1,202 (48.3%) and 1, 278 (51.7%) students from urban and rural areas, respectively. Additionally, 870 (35%) were only children, while 1,619 (65%) had siblings. There were 428 (17.2%) migrant children and 2,061 (82.8%) non-migrant children.

**Table 1:** The results of demographic characteristics analysis (*n* = 2,489).

Demographic characteristics	Items	*n*	%
Gender	Boy	1,232	49.5
	Girl	1,257	50.5
Grade	Grade 7	593	23.8
	Grade 8	561	22.5
	Grade 9	568	22.8
	Grade 10	282	11.3
	Grade 11	247	9.9
	Grade 12	238	9.6
Residence	Rural	1,287	51.7
	Urban	1,202	48.3
Being an only child?	Yes	870	35
	No	1,619	65
Being anMigrant child?	Yes	2061	82.8
	No	428	17.2

### 3.2 Correlation analysis

Table 2 presents the correlation test results for the key variables involved in this study, showing significant correlations among them. Specifically, SC was significantly negatively correlated with AA (*r* = −0.440, *p* < 0.01) and with MPA (*r* = −0.688, *p* < 0.01), H1 and H2 were supported. AA positively correlated with MPA (*r* = 0.415, *p* < 0.01). This indicates that higher SC was associated with lower AA and MPA, whereas higher AA was associated with higher MPA.

**Table 2:** The results of correlation analysis.

Variables	*M*	SD	1	2	3
1. Self-control	3.07	0.64	1		
2. Academic anxiety	3.23	0.89	−0.440^**^	1	
3. Mobile phone addiction	2.77	0.99	−0.688^**^	0.415^**^	1

^**^p < 0.01.

### 3.3 Mediation effect analysis

**Figure 2:**
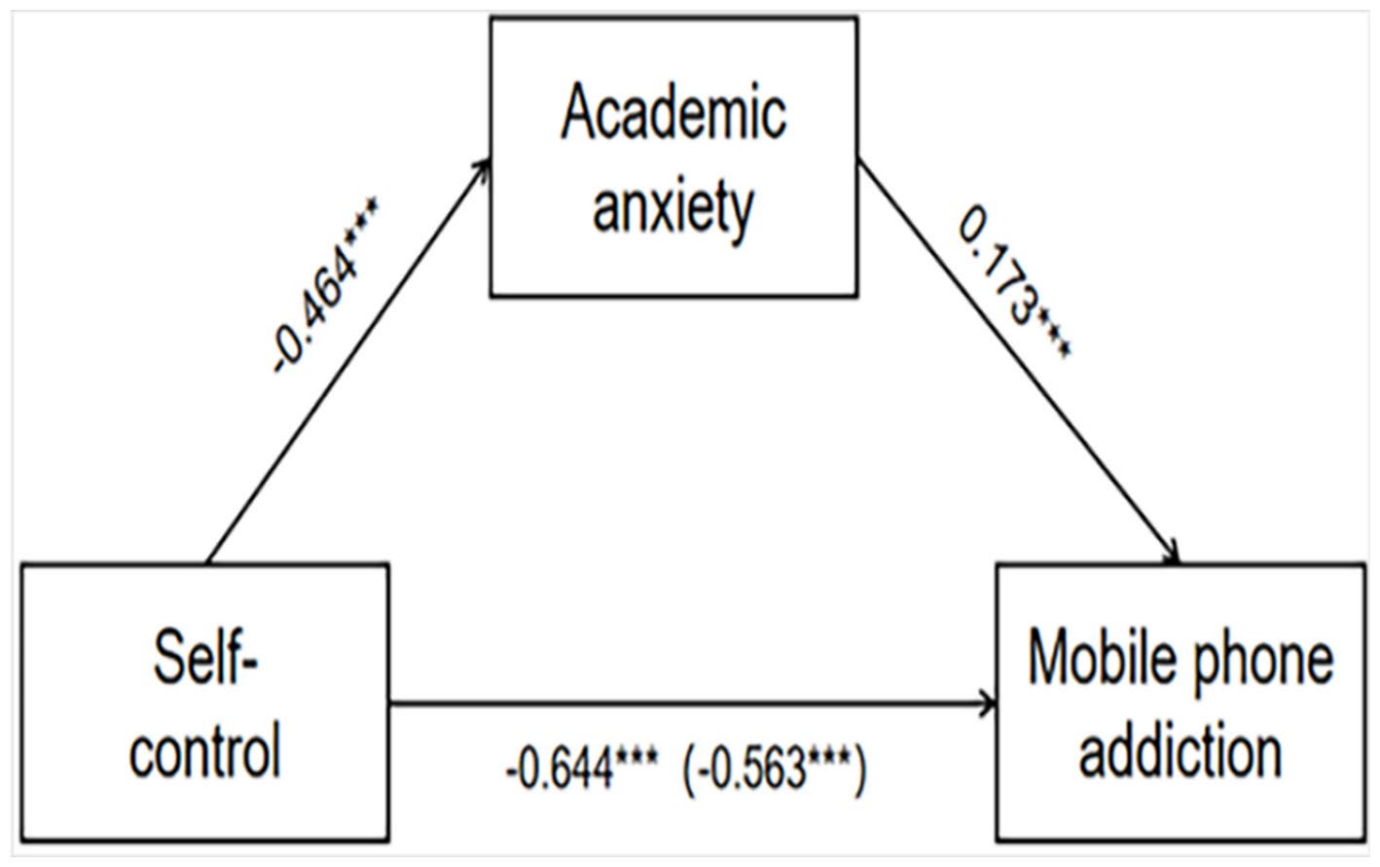
The mediation effect analysis (***p < 0.001).

As presented in Figure 2, which illustrates the path diagram of the mediation effect analysis, the total effects of SC on MPA (*β* = −0.644, SE = 0.022, *p* < 0.001) were significant. The direct effect of SC on MPA (*β* = −0.563, SE = 0.024, *p* < 0.001) also was significant. Additionally, SC significantly negatively predicts AA (*β* = −0.464, SE = 0.026, *p* < 0.001), and AA significantly positively predicts MPA (*β* = 0.173, SE = 0.017, *p* < 0.001). Furthermore, there is compelling evidence of a significant mediating link because the 95% CIs for the indirect effect of SC on MPA through academic anxiety [−0.10, −0.06] did not contain zero, H3 was supported.

### 3.4 Moderation effect analysis

The PROCESS 4.3 (Model 14), was employed to verify the moderating effect of being an only child between AA and MPA. The analysis indicated that the factor of “being an only child” moderates the relationship between AA and MPA (*p* < 0.001), H5 was supported.

Drawing 5,000 samples to estimate the bootstrap 95% confidence interval. All variables were standardized, and sex, grade, residence, and whether the child was a migrant were controlled. Table 3 presents the results of the moderation effect analysis, the results indicated that SC had a significant negative predictive effect on MPA (*β* = −0.56, *p* < 0.001, SE = 0.02) and AA (*β* = −0.46, *p* < 0.001, SE = 0.03). Additionally, AA was a significant positive predictor of MPA (*β* = 0.18, *p* < 0.001, SE = 0.03). Being an only child also had a significant negative predictive effect on MPA (*β* = −0.29, *p* < 0.001, SE = 0.03), H4 was supported. Furthermore, the interaction between being an only child and AA significantly predicted MPA (*β* = −0.13, *p* < 0.001, SE = 0.03), indicating that being an only child had a significant moderating effect.

**Table 3:** The results of moderation effect analysis (*n* = 2,489).

Predict variables	Results variables					
	MPA			AA		
	*β*	*t*	95%CI.	*β*	*t*	95%CI.
Self-control	−0.56	−35.79^***^	[−0.59, −0.53]	−0.46	−25.22^***^	[−0.50, −0.43]
Academic anxiety	0.18	11.85^***^	[0.15, 0.21]			
Being an only child	−0.29	−9.80^***^	[−0.34, −0.23]			
Being an only child × Academic anxiety	−0.13	−4.57^***^	[−0.19, −0.08]			
*R^2^*	0.552			0.206		
*F*	382.45^***^			128.87^***^		

Sex was dummy-coded such that 0 = men and 1 = woman; address was dummy-coded such that 0 = rural and 1 = urban; migrant status was dummy-coded such that 0 = yes and 1 = no; grade was a dummy variable, with senior high School and junior high school as reference variables.

***p < 0.001.

**Figure 3:**
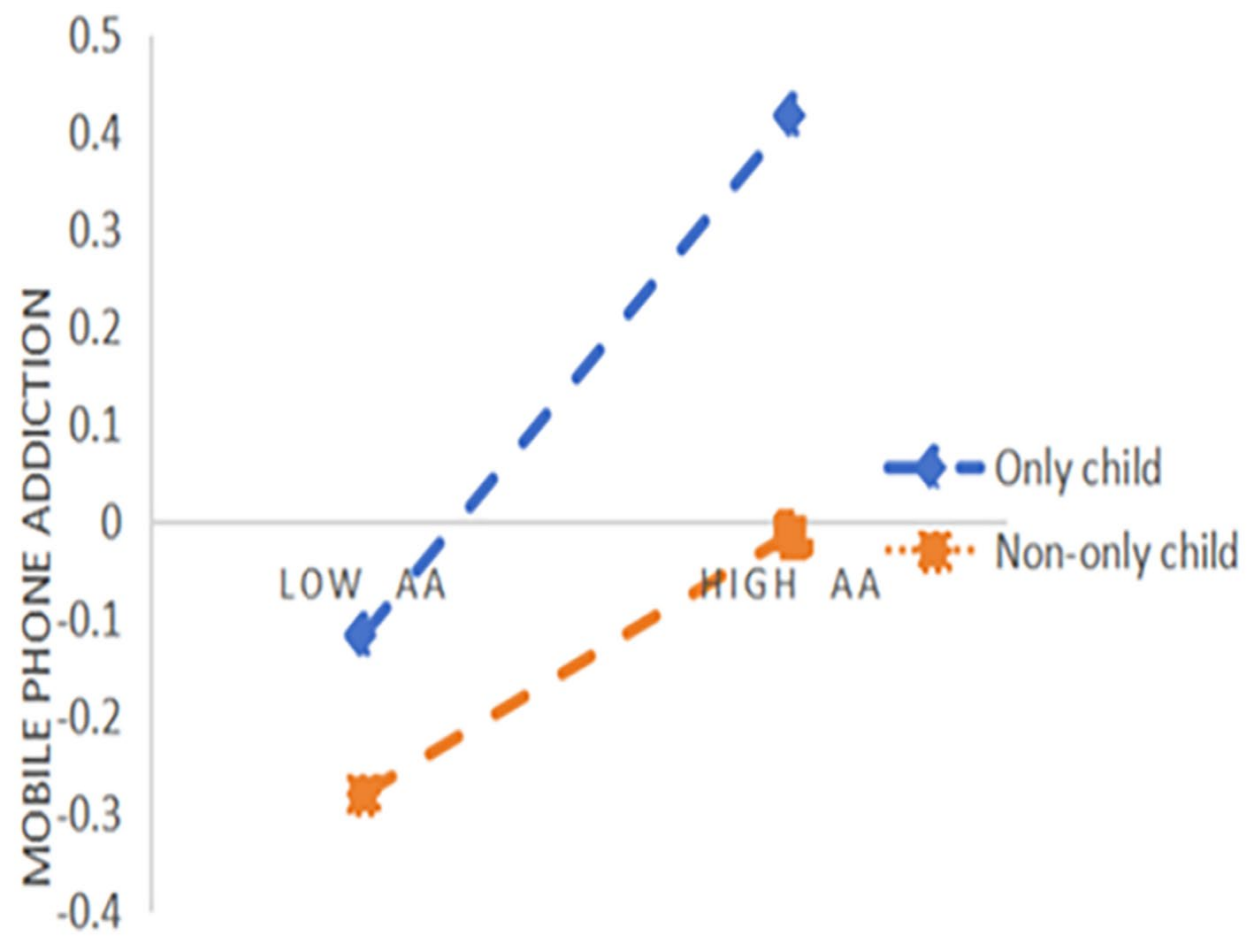
Simple slope effect.

Table 4 presents the results of the moderating effect of only child and non-only child, as well as the indirect effects of SC on inhibiting MPA by improving AA among only child and non-only child. The result indicated that for being an only child (M − 1 SD), AA had a significant positive predictive effect on MPA with regression coefficients of 0.27, *t* = 10.5, *p* < 0.001, respectively. For non-only child (M + 1SD), although AA positively predicted MPA, its predictive effect was smaller, with a regression coefficient of 0.13, *t* = 7.66, *p* < 0.001. This indicates that, compared to only child, the predictive effect of AA on MPA was significantly reduced for non-only child. Figure 3 shows the simple slope analysis diagram, at both the levels of only and non-only child, the mediating effect of AA between SC and MAP showed a decreasing trend. This means that, compared to non-only child, only child are more likely to reduce their AA and subsequently curb their MPA through interventions that enhance SC.

**Table 4:** The direct and indirect effects of the moderated mediation effect.

Types of effects	Being an only child	*β*	Boot. SE	Boot. CI	Boot. UI
Direct effect	−0.65(M − 1SD)	0.27	0.03	0.22	0.32
	0.35(M + 1SD)	0.13	0.02	0.10	0.17
Indirect effect	−0.65(M − 1SD)	−0.12	0.01	−0.15	−0.10
	0.35(M + 1SD)	−0.06	0.01	−0.08	−0.04

## 4 Discussion

This study aimed to examine the correlation between SC, AA, MPA, and being an only child in middle school students and to test the moderating effects of being an only child on these factors. These results indicate that SC has a direct and significant adverse predictive effect on MPA. Additionally, a significant mediating relationship was identified: SC negatively impacted AA, which positively impacted MPA. Being an only child emerged as an essential moderator in the relationship between AA and MPA, with only children exhibiting a strong positive impacted effect of AA on MPA. This finding suggests that only children may be better equipped to manage AA and reduce MPA through SC. These findings highlight the combined influence of personal and familial factors on MPA and emphasize the moderating role of being an only child status, thereby addressing the gaps in previous research.

This study indicates that SC significantly impact MPA. Students with high SC can reduce the occurrence of MPA behavior (Duckworth et al., 2019). Individuals with high SC abilities can better manage their cognitive and emotional responses to AA (Jiang and Zhao, 2016) and are more inclined to engage in activities that offer long-term rewards and satisfaction. On the contrary, those with low SC prefer activities that provide immediate pleasure (John et al., 2020). Therefore, students with higher SC are more likely to employ positive self-regulation strategies rather than seeking instant gratification from smartphones, thereby reducing the likelihood of MPA (Paschke et al., 2016; Gollwitzer, 1999). Enhancing middle school students’ SC is crucial for mitigating MPA. Research indicates that practicing goal-setting and linking personal plans to situational cues (e.g., ‘When I enter my bedroom, I will study math’) helps students focus on situational features and establish strong psychological connections with expected behaviors (Galla and Duckworth, 2015), thus reinforcing their SC abilities. Additionally, establishing personal rules and cultivating good study habits (such as studying at the same time and place every day) can strengthen specific behaviors and enhance SC (Kardefelt, 2014), thereby reducing the incidence of MPA.

This study indicates that AA mediates the relationship between SC and MPA. Self-regulation theory posits that individuals can regulate their thoughts, emotions, and behaviors through setting goals, monitoring actions, and adjusting strategies to adapt to environmental demands and achieve personal objectives (Bandura, 1991). Research indicates that individuals experiencing abnormal emotions such as AA may use smartphones as a coping strategy to regulate negative emotions, resulting in positive emotional experiences (Markus et al., 2009). When this sense of satisfaction is positively associated with smartphone use, individuals may rely on smartphone usage to control anxiety, leading to MPA (Hou et al., 2021). However, individuals with strong SC abilities perform better in active emotional regulation tasks. Firstly, students with high SC abilities tend to choose more positive emotional regulation strategies (such as planning to improve the situation or viewing it more positively) when dealing with AA, thereby reducing the occurrence of MPA (Gollwitzer, 1999). Secondly, students with high SC abilities might manage their cognition and expectations, reducing the interference of AA (Wang et al., 2020), thus decreasing the risk of MPA to some extent. To mitigate the impact of AA on SC and MPA, it is essential for schools, families, and students to avoid setting excessively high academic goals or expectations, which can exacerbate academic pressure and anxiety. Additionally, fostering academic resilience in students can enhance their ability to cope with academic setbacks and negative emotions, thereby reducing the influence of AA on MPA (Quach et al., 2015).

This study also found that being an only child significantly moderated the relationship between AA and MPA. According to family systems theory, the interactions among various subsystems within the family collectively influence individual development (Martha and Blair, 1997). In the family systems theory proposed by American scholar M. Bowen, the family is conceptualized as a “group emotional unit,” which emerges from the interactions within the father–mother–child triangle, where individuality (separation) and solidarity (fusion) are inherently intertwined. Individuality entails the pursuit of self-independent identity, whereas solidarity reflects the expectation of group belonging. The imbalance between these two dynamics can lead to dysfunction in the emotional management system of the nuclear family (Rasmussen, 2017). Within the broader family context, individuals are relatively autonomous. However, when children are compelled to consistently attend to parental demands and emotional expressions, they may lose their autonomy, becoming unable to think and act according to their own needs. Sustained exposure to such conditions leads to chronic psychological tension, thereby escalating emotional stress (Guan et al., 2018), turning to Smartphones for a sense of belonging. Chinese society is characterized by a cultural emphasis on “making the child successful.” Most Chinese parents tend to expect their children to receive better education and achieve higher academic performance, thereby securing a better future. On one hand, in one-child families, children often become the sole focus of family attention, increasing the family’s emphasis on academic performance (Quach et al., 2015). Excessive attention and educational investment make students more prone to AA (Liu et al., 2005). To cope with this anxiety, they may alleviate negative emotions by using smartphones (Kardefelt, 2014), which can easily lead to addictive behavior. On the other hand, Positive sibling relationships can divert family attention and dilute family resources, thereby mitigating the impact of anxiety and reducing maladaptive behaviors (Gass et al., 2007). Siblings in non-single-child families can provide each other with emotional care and encouragement, thus relying less on external electronic media to compensate for the missing emotional belongingness from parents when facing AA. Conversely, only children, lacking direct emotional support and companionship from siblings during AA, tend to seek external emotional comfort and distraction through playing with their phones. Conversely, non-only children benefit from Sibling support, enabling them to relieve anxiety through emotional support and mutual assistance rather than over-reliance on smartphones, which helps reduce stress and anxiety (McHale et al., 2009). Additionally, the study found that only children more strongly weaken AA through enhancing self-control and thereby inhibit MPA compared to non-only children. This may be due to the fact that excessive parental intervention weakens students’ intrinsic motivation and hinders the development of their self-control ability (Li et al., 2019). Given that most Chinese middle school students are only children, parents should provide children with certain autonomy, adopt a relaxed and democratic parenting style instead of an intrusive one, and focus on enhancing children’s SC to promote their physical and mental health and reduce addictive behaviors (Nie et al., 2022).

## 5 Conclusion

This study constructs a research model by adopting the self-regulation theory and the family systems theory. The research findings indicate that self-control can reduce mobile phone addiction. The self-control of adolescents can indirectly reduce their mobile phone addiction by alleviating their academic anxiety. Compared with adolescents with siblings, only children are more likely to develop higher levels of mobile phone addiction due to academic anxiety. This study makes contributions in both theoretical and practical aspects. Theoretically, this study constructs a “self-control-academic anxiety-mobile phone addiction” model to explain how self-control reduces adolescents’ mobile phone addiction by alleviating their academic anxiety. On the one hand, this model is based on and expands two classic theories, namely the self-regulation theory, and the family systems theory. On the other hand, it provides a new theoretical framework for studying the problematic mobile phone use of only children and non-only children caused by negative academic emotions.

In terms of practical significance, this study emphasizes the strategies for school teachers to cultivate students’ self-control ability during the teaching process. Teachers can enhance students’ self-control by setting relevant goals, formulating personal rules, and cultivating good study habits. Meanwhile, in terms of family education, this study highlights the importance of healthy media use habits and family parenting styles at home. We encourage the formulation of family media use rules and advocate adopting a relaxed and democratic parenting style to improve adolescents’ self-control and relieve their academic anxiety, so as to ensure the physical and mental health development of adolescents. In conclusion, this moderated mediation model offers a new perspective for understanding how adolescents’ self-control inhibits mobile phone addiction by relieving academic anxiety and for exploring the reasons for the differences in the levels of mobile phone addiction between only children and non-only children.

## 6 Limitations

This study presents several notable limitations. The cross-sectional design fails to capture the dynamic evolution of variables during adolescents’ critical developmental period, thus inadequately reflecting temporal changes in the research model. Furthermore, the reliance on self-report measurements introduces potential biases: respondents may underreport addiction symptoms to conform with social expectations, memory limitations may compromise accurate recall of usage patterns, the methodology cannot account for contextual variations in smartphone use, and the absence of objective metrics such as screen time data constrains validation of self-reported information. Future investigations would benefit from implementing more stringent random sampling approaches, employing quasi-experimental designs for intervention research, conducting longitudinal studies to examine time-series variations, and utilizing diverse data collection methodologies to mitigate self-reporting biases.

## Data Availability

The original contributions presented in the study are included in the article/supplementary material, further inquiries can be directed to the corresponding author.

## Footnotes

^1^ https://www.wjx.cn/
